# The Need to Enhance the Use of Teleconsultation: A Qualitative Study Among Health Providers to Reduce the Burden of Low Birth Weight

**DOI:** 10.1155/ijta/2336484

**Published:** 2025-07-14

**Authors:** Wiwin Lismidiati, Widyawati Widyawati, Elsi D. Hapsari, Akhmadi Akhmadi, Dimas S. E. W. Sumunar

**Affiliations:** ^1^Department of Pediatric and Maternity Nursing, Faculty of Medicine, Public Health, and Nursing, Universitas Gadjah Mada, Yogyakarta, Indonesia; ^2^Department of Psychiatric and Community Nursing, Faculty of Medicine, Public Health, and Nursing, Universitas Gadjah Mada, Yogyakarta, Indonesia; ^3^Department of Health Policy and Management, Faculty of Medicine, Public Health, and Nursing, Universitas Gadjah Mada, Yogyakarta, Indonesia

**Keywords:** digital technology, low birth weight, mobile health, mother, pregnancy

## Abstract

**Background:** Low birth weight remains a health concern in the Special Region of Yogyakarta, Indonesia. Effective management of low birth weight cases is crucial, yet community-based health programs face challenges in addressing this issue.

**Purpose:** This study is aimed at exploring the needs, experience, and perspectives of healthcare providers regarding low birth weight prevention programs.

**Methods:** This was a qualitative descriptive study. Data were collected from June to December 2023 in Kulon Progo District, Yogyakarta, Indonesia. Data were collected through a focus group discussion with nine midwives. Triangulation was conducted with 12 breastfeeding mothers, one nutritionist, and one head of the primary health center through in-depth interviews. Each discussion and interview were recorded, transcribed, and subjected to thematic analysis to identify key themes.

**Results:** Three themes were discovered in this study: programmatic initiatives of healthcare providers; poor knowledge, attitude, and practice with limited resources and access; enhancing the use of teleconsultations. Programmatic initiatives from primary healthcare centers have focused on preventing low birth weight through targeted outreach, education, and prenatal care for at-risk mothers. However, the persistent challenge of insufficient human resources for health has hindered the effectiveness and reach of these efforts. To enhance LBW prevention strategies, the needs of digital technology by integrating digital tools and electronic health records are imperative.

**Conclusion:** Effectively managing low birth weight requires utilization of technology to strengthen healthcare interventions. Digital solutions enhance communication, broaden service reach, and enable timely support, especially in underserved areas. Incorporating these tools into health programs improves the delivery and accessibility of care, presenting a valuable approach to advancing maternal and child health outcomes.

## 1. Introduction

Low birth weight (LBW) is a leading contributor to infant mortality, particularly in the newborn period, with LBW infants facing a 20-fold increased risk of death compared to those with normal birth weight [1]. According to the 2023 Indonesian Health Survey, 23.6% of LBW infants did not receive specialized treatment, highlighting significant gaps in care [2]. The prevalence of LBW in Indonesia showed a slight decrease during the period 2022–2024, with the percentage of mothers who gave birth to liveborn infants with LBW in the past 2 years declining from 12.58% in 2022 to 12.54% in 2023 and 12.47% in 2024 [3]. Various maternal and socioeconomic factors contribute to the high incidence of LBW in Indonesia.

LBW is prone to cause serious health problems for some infants. The following symptoms are frequently experienced by LBW babies: respiratory distress, hypothermia, feeding difficulties, and risk for infection. Long-term developmental problems including delayed growth, illnesses, and neurological diseases are also attributed to LBW, which may cause neonatal and post-neonatal mortality. LBW can also lead to hypoglycemia, hypocalcemia, and polycythemia in the early neonatal period, which demands comprehensive healthcare interventions [4, 5].

Effective management of LBW cases requires a healthcare system that is both comprehensive and accessible, yet this is often difficult to achieve, particularly in resource-limited settings [6]. Barriers such as inadequate staffing, limited access to advanced care, and geographic obstacles further complicate the delivery of necessary interventions. Additionally, high costs associated with specialized care can place a heavy financial burden on families, reducing their access to the support these infants need. Addressing these challenges requires resource-sensitive solutions that ensure LBW infants receive timely and effective care, ultimately helping to reduce infant morbidity and mortality rates in underserved communities [7].

Globally, extensive efforts have been dedicated to preventing LBW, with initiatives focused on enhancing maternal health, nutrition, and access to prenatal care [8, 9]. The World Health Organization (WHO) underscores the importance of comprehensive and preventive healthcare approaches, emphasizing the need for early maternal support and the integration of community-based programs to improve maternal and neonatal outcomes [10]. Many regions have adopted these guidelines to fit local contexts, implementing strategies that include digital health technologies, outreach programs, and coordinated care services to improve outcomes for mothers and infants. In Indonesia, efforts to address LBW are increasingly focused on community health outreach and digital tools designed to extend healthcare access and enable early identification and support for at-risk pregnancies [11]. These initiatives are collectively aimed at building a supportive healthcare environment that prioritizes early intervention, continuity of care, and accessibility for mothers across diverse settings.

In the Special Region of Yogyakarta, numerous initiatives have been introduced to prevent LBW, incorporating digital tools such as web-based applications and electronic health records to enhance patient monitoring and streamline care. These technologies are intended to improve communication between healthcare providers and patients, facilitate early intervention, and ensure consistent follow-up. Primary care centers have also conducted outreach, focusing on at-risk mothers by offering education, nutritional guidance, prenatal care, and resources designed to reduce LBW incidence. Through these efforts, primary care centers aim to support maternal health and foster a community-based approach to LBW prevention.

Kulon Progo is one of the districts which is located in the west part of Yogyakarta with a specific features regarding the nature and community characteristics. It has a plateau and Menoreh Hill in the north part with 500–1000 m above sea level and low plains in the south part with 0–100 m above sea level. Kulon Progo district plays a role as a basic farming commodity supported by tourism, mining, and the maritime industry. The regency covers an area of 586.28 km^2^ with approximately 438.644 inhabitants [12].

Between 2020 and 2024, Kulon Progo Regency experienced a consistent decline in live births, from 41,030 to 32,455. During this period, infant mortality showed a generally downward trend, falling from 38 deaths in 2020 to 35 in 2024, despite a notable peak of 56 in 2021. These figures reflect gradual improvements in neonatal care and child health services, although the fluctuations suggest that sustained efforts are needed to ensure consistent quality and accessibility of care across the region. In 2023, there were 308 LBW cases out of 3944 live births in Kulon Progo, accounting for 7.81%. This percentage exceeds the national target of less than 5% and continues a trend of fluctuating LBW rates above 6% since 2015 [13].

Maternal mortality during the same 5-year span showed significant year-to-year variation. There were three maternal deaths in 2020, rising sharply to 10 in 2021, before declining to eight in 2022, dropping further to just one in 2023, and slightly increasing again to four in 2024 [13]. These changes, set against the backdrop of declining birth rates, highlight ongoing challenges in maternal health, particularly in managing complications during pregnancy, childbirth, and the postpartum period.

Kulon Progo Regency in Yogyakarta is served by a diverse network of health facilities that support maternal and neonatal care. The district comprises two publicly owned hospitals, as well as 21 government-managed community health centers. In addition, the private sector contributes with 7 general hospitals, 97 individual doctor practices, 2 blood transfusion units, 2 health laboratories, and 69 pharmacies. The availability of specialized units such as neonatal intensive care units (NICUs) within hospital infrastructure ensures critical care for newborns requiring advanced medical attention [13]. In Kulon Progo Regency, five out of nine hospitals are equipped with NICU facilities, offering a total of 17 beds across the region. The largest hospital accounts for the majority, with 10 NICU beds.

The human resource capacity in Kulon Progo Regency is critical to supporting maternal and neonatal health services. As of 2023, the district is served by a total of 145 medical specialists, 134 general practitioners, 41 dentists, 333 midwives, 880 nurses, 150 pharmaceutical staff including pharmacists, 39 public health professionals, 49 environmental health workers, and 59 nutritionists and dietitians [13].

To better understand and address these limitations, it is essential to explore the perspectives of healthcare providers directly involved in LBW care. Their insights into the barriers and enablers of current approaches could provide valuable guidance for refining strategies, improving maternal and infant health outcomes, and achieving long-term success in LBW prevention efforts.

## 2. Materials and Methods

This study employed a qualitative descriptive design to explore the perspectives of healthcare providers directly involved in the care of LBW infants, with a focus on identifying their needs regarding LBW prevention programs. Participants included nine midwives working at a public health center in Kulon Progo District. Data collection took place from June to December 2023. Inclusion criteria specified healthcare providers from primary healthcare centers who were actively engaged in maternal and child health services. To ensure data relevance and accuracy, individuals not directly involved in such care—such as administrative staff or professionals from unrelated specialties—were excluded. Gestational age was not used as a criterion; thus, both preterm and term LBW cases were included.

To support data triangulation, mothers and primary care managers were also included. Mothers were recruited from four selected villages and were eligible if they had delivered within the past 12 months, resided in the designated area, and provided informed consent. The sample comprised eight mothers who had delivered LBW infants and four who had delivered infants with normal birth weight, enabling comparative insights. Mothers were excluded if their infants had congenital anomalies or serious health complications, if they had relocated, or if they declined participation or provided incomplete or unreliable data. Additionally, one primary care manager and one nutritionist were included to broaden the perspective and validate findings from multiple professional roles.

In this study, mothers who delivered LBW babies were included with specific exclusion criteria applied to births involving congenital birth defects or significant complications that could independently affect neonatal outcomes. Gestational age was not used as an explicit criterion for inclusion or exclusion; therefore, both preterm and term LBW infants were considered within the scope of the study.

Data collection involved both focus group discussions (FGDs) and in-depth interviews to obtain comprehensive insights. An FGD was conducted by a member of the research team with support from two research assistants at a primary healthcare center. The session, lasting approximately 45 min, took place in a private room to ensure confidentiality and was audio-recorded. An interview guide structured the discussion, and field notes were taken to support the analysis. In-depth interviews were primarily conducted at primary healthcare centers, with three of the 12 participating mothers interviewed at home due to mobility constraints and lack of transportation. Prior to each interview, participants were provided with a clear explanation of the study objectives to ensure informed and voluntary participation; all invited individuals consented to take part.

An interview guide was developed to examine healthcare providers' perspectives and experiences in managing LBW cases at Kalibawang Health Center. Additionally, a separate guide was prepared for mothers to capture their experiences in caring for their children. To ensure consistency in data collection, both LBW and non-LBW groups were interviewed using a shared thematic framework. Core topics included participants' educational and professional backgrounds, case descriptions, implemented interventions, encountered challenges, facilitating factors, stakeholder engagement, unmet needs, maternal risk factors, program evaluation, and proposed innovations. The research questions were formulated based on a comprehensive review of relevant literature and aligned with the study's objectives. To ensure content validity and relevance, the questions were subsequently reviewed and refined by a panel of experts in maternal and child health.

Prior to data collection, the FGD guides and questions for both health professionals and mothers underwent a thorough review by a panel of experts in maternal and child health to ensure clarity, relevance, and appropriateness. Although no formal pilot test was conducted, this expert validation served as an essential step in refining the questions. For source triangulation with medical doctor and nutritionist, questions were specific to their roles and perspectives within maternal and neonatal care based on the findings from FGD with midwives.

Triangulation in this study was carried out using both methodological and source triangulation to enhance the credibility of the findings. Methodological triangulation was achieved through the combination of in-depth interviews and direct observation. Source triangulation involved engaging participants from diverse professional backgrounds—including mothers, a nutritionist, and a medical doctor—to validate and enrich the findings derived from the FGDs. In total, in-depth interviews were conducted with 12 mothers, one nutritionist, and the head of the public health center. The source triangulation process was carried out by two members of the research team alongside one trained research assistant.

This study employed a content analysis approach to systematically analyze the qualitative data, utilizing coding and theme identification to uncover patterns within participants' responses. The analysis process began with each author independently coding the transcripts, ensuring that initial interpretations were closely tied to the raw data. After this independent phase, the research team convened regularly to compare findings, discuss emerging themes, and refine the coding framework. These collaborative meetings enabled the identification of consistent patterns and supported the development of a coherent thematic structure. Discrepancies between individual codes were resolved through discussion and consensus, with the team collectively adjusting the coding scheme as needed.

Ethical permission was granted from the Faculty of Medicine, Public Health, and Nursing Universitas Gadjah Mada ethical committee prior to data collection, number KE-FK-1194-EC-2023 on July 18th 2023.

## 3. Results

Characteristics of the participants were displayed in Tables 1 and 2. There were nine midwives between 37 and 54 years old who participated in this study. All of them were midwives with 3-year diplomas. The range of their experience of working as midwives was between 15 and 33 years (Table 1). The characteristics of the medical doctor and nutritionist in triangulation could be seen in Table 2. The medical doctor has 8 years of experience, and the nutritionist has 30 years of experience. Among mothers who were interviewed in triangulation, most of them had an educational background of senior high school (83.33%). The range of mother's age was between 16 and 40 years old (see Table 3 for details).

This study identified three overarching themes: programmatic initiatives of the healthcare providers; poor knowledge, attitude, and practice with limited resources and access; and enhancing the use of teleconsultations. The development of these themes was guided by a structured process involving the organization of raw data into codes, which were then grouped into subthemes before being refined into final themes. The detailed progression from codes to subthemes and ultimately to themes is presented in Table 4.

### 3.1. Theme 1: Programmatic Initiatives of the Healthcare Providers

Healthcare providers play a crucial role in delivering quality care through various structured programs aimed at enhancing the health and well-being of the population. These initiatives are designed to address specific health needs and to ensure that healthcare services are accessible, comprehensive, and patient-centered. The programs often involve a multidisciplinary approach, leveraging the expertise of doctors, nurses, dietitians, and other health professionals from the primary health center to provide holistic care experience. The ultimate goal of these initiatives is to improve health outcomes, reduce health disparities, and promote sustainable health practices within communities.

#### 3.1.1. Home Visits

Home visits are a pivotal component of healthcare services that bring medical and supportive care directly to the vicinity of patients, making them especially beneficial for vulnerable populations such as mothers and LBW infants. This initiative is crucial for new mothers who may face challenges in accessing healthcare facilities due to mobility issues, lack of transportation, or the demands of caring for a newborn. During home visits, healthcare providers, including nurses and community health workers, conduct routine check-ups, provide education on infant care, and develop personalized care plans tailored to the needs of both the mother and the LBW infant. By addressing health concerns in the comfort of the patient's home, these visits aim to enhance maternal and infant comfort, ensure continuous care, and reduce the risk of complications that can lead to hospital readmissions. Ultimately, this approach supports the overarching goal of improving health outcomes for LBW infants and promoting maternal well-being through accessible, individualized care.

*Home visit intended for mother with high-risk pregnancy, including caesarean history or with small mid upper arm circumference, chronic energy deficiency and lack of weight gain” (HC5)*

This finding was confirmed by mothers who also mentioned that home visits are helpful:

*“The midwife, during the prenatal period also visits pregnant women..... in addition for malnutrition toddlers, meal supplements is provided” (M11)*

#### 3.1.2. Dietary Support

Dietary support is a critical aspect of preventive and therapeutic healthcare, particularly in the context of LBW prevention and management. This initiative emphasizes the importance of personalized nutrition counseling and diet plans for pregnant women and new mothers, as well as for infants at risk of LBW. Healthcare providers, including dietitians and nutritionists, collaborate with mothers to assess their dietary habits, educate them on the nutritional needs essential for fetal and infant growth, and develop tailored nutrition strategies that align with their health goals. By integrating dietary support into LBW prevention programs, the initiative seeks to enhance maternal nutrition, promote healthy weight gain during pregnancy, and ultimately improve the health outcomes of infants. This approach not only empowers mothers to make informed food choices but also addresses the underlying nutritional factors that contribute to LBW, thereby fostering a healthier start for newborns and reducing the incidence of related complications.

*Meal supplement program is a multistakeholder approach, involving primary health care and the local government (HC1)*

One participant highlighted the role of community resources in dietary support, stating the following:

*(The meal supplements) from the village. If it's from what's the primary health care, sometimes they're invited there to collect it. (M11)*

#### 3.1.3. Integrated Health Post

Integrated health posts are community-based healthcare facilities that play a crucial role in addressing LBW by providing a comprehensive range of health services under one roof. Staffed by multidisciplinary teams, including doctors, nurses, and midwives, these posts offer coordinated maternal and child health programs, nutrition counseling, immunizations, and health education. This integrated approach ensures that mothers receive essential support throughout their pregnancy and postpartum period, particularly in rural or underserved areas where access to healthcare can be limited. By facilitating access to vital services, integrated health posts contribute significantly to the prevention and management of LBW cases.

*The mothers rarely understand about the essence of adequate nutrition despite anytime they attend for checkups or at the Posyandu we have delivered the educational materials (HC5)*

That finding confirmed with what explained by mothers as follows:

*[The midwives] often at the Posyandu (health post), recommend me to eat and consume [healthy] foods. (M2)*

*In my opinion, the educational session is adequate. [The midwives] told me how to breastfeed well, during the pregnancy class, like that. It was very helpful for caring for the baby, especially every time we come home, pregnant women are given something like vegetables, fruit, side dishes (M3)*

#### 3.1.4. Private Consultations

Private consultations provide an essential avenue for mothers of LBW infants to receive personalized medical advice and treatment in a confidential setting with healthcare providers. This initiative is designed to give mothers the privacy and individualized attention they need to discuss their health concerns and the specific needs of their LBW infants in detail. Private consultations can take place in clinics, hospitals, or through telemedicine platforms, depending on patient preferences. The primary aim is to foster a trusting relationship between the mother and healthcare provider, ensuring that care is tailored to the unique medical history and current health status of both the mother and her LBW infant.

*“There are some mothers who are unskillful in using the technology but they poses ownership of Android smartphone and operates WhatsApp in daily basis. Therefore, we taught them to take benefit (of the application), even we support their internet connection for real-time self-reporting” (HC5)*

That finding is confirmed by one participant as follows:

*For that case [verifying reliability of the sources], I usually ask the midwife, since they already knows more. They have interact with so many patients before, so I could learn more…. I frequently ask a question to the midwife, since she is the one who understand and has been taking care a lot of people (M11)*

#### 3.1.5. Targeted Interventions

Targeted health interventions can play a crucial role in addressing the specific needs of patients at risk for LBW by offering personalized care and support tailored to their unique circumstances. The study highlighted how these interventions can effectively address specific health concerns associated with LBW, such as nutritional deficits and maternal health issues. By promoting healthy behaviors and providing relevant education, targeted interventions can lead to improved health outcomes for both mothers and infants.

*The focus is more on children who are at risk of stunting so they are not yet stunted, but how come their weight doesn't increase for several months, so that's what needs immediate intervention (HC1)*

Mothers confirmed their interaction with the care providers:

*(I receive support) when I attend primary health center, in addition to outreach visits by the midwives at home. Dietary package for children also provided (M10)*

### 3.2. Theme 2: Poor Knowledge, Attitude, and Practice With Limited Resources and Access

#### 3.2.1. Poor Mothers' Nutritional Status

Pregnancy-related weight gain is a critical factor influencing the risk of LBW. Participants assumed that eating difficulties are a habit since teenage years. On the other hand, based on the results of triangulation, it was found that women's experiences highlight how personal and societal attitudes, motivations, and beliefs shape their approaches to weight management during pregnancy. Some women actively encouraged healthy weight gain practices, recognizing the importance of maintaining a healthy weight for both themselves and their infants. However, women also noted that their eating habits were influenced by psychological states, with some linking emotional eating to distress, while others found that exercise and a nutritious diet helped manage stress and anxiety. Conversely, weight gain and changes in body image heightened anxiety for some, potentially leading to unhealthy eating behaviors. These psychological dynamics are crucial to consider in LBW prevention strategies, as they significantly impact maternal health and birth outcomes.

*[We assume, eating difficulties] is a habit, so since teenagers they had eating difficulties and proceed to pregnancy and breastfeeding eating was difficult for them (HC8)*

*Eating difficulties often not only in the first trimester, but particularly that type of person with chronic energy deficiency are lazy to eat. (HC9)*

The mothers were aware about the needs of adequate nutrition during pregnancy:

*(To mitigate), the dietary pattern should shift to high protein and vegetables (M5)*

#### 3.2.2. Lack of Knowledge

Participants in this study identified ignorance as a significant barrier to achieving healthy weight gain during pregnancy, which is critical in preventing LBW. One of them said that family's parenting style and mothers' educational background are contributing factors for incapabilities. Based on the triangulation with the mother, numerous participants reported challenges in accessing reliable information regarding appropriate weight gain and dietary practices during pregnancy. They expressed frustration with the information provided, describing it as either completely omitted, overly vague, inconsistent, or lacking relevance. Additionally, when participants attempted to discuss weight gain during prenatal visits, they often felt ignored by their healthcare providers. Some participants voiced dissatisfaction with the formats in which information was presented, such as preferring personal conversations over printed materials. Moreover, several participants indicated a need for more practical resources, such as recipes and actionable suggestions. There was a clear desire among participants for their healthcare providers to offer more comprehensive information, engage in regular discussions, and provide ongoing support, as these elements are essential for managing weight gain effectively and mitigating the risk of LBW.

*Family's parenting style and mothers' educational background are contributing factors for incapabilities (HC11)*

Mothers confirmed that they are actively searching for health resources:

*I was immediately search from Google about growth and development, medication for rash, developmental milestones for 2 months old baby. (M8)*

#### 3.2.3. Pregnancy Complications

Participants in this study identified that pregnancy complications in their district were dominated by prematurity, gemelli, and severe preeclampsia.

*Pregnancy complications in our district dominated by prematurity, gemelli and severe preeclampsia (HC8)*

*Premature birth, many of them having pregnancy complications due to hypertension and pre-eclampsia (HC5)*

#### 3.2.4. Passive Smoking

Care providers have observed that pregnant women exposed to secondhand smoke are at a higher risk of having LBW infants. They have noted that exposure to secondhand smoke can negatively impact fetal growth, leading to LBW and premature delivery. Healthcare providers recognize that the harmful effects of nicotine and other chemicals in tobacco smoke contribute significantly to these adverse outcomes. As a result, care providers emphasize the importance of pregnant women avoiding exposure to secondhand smoke and refraining from smoking behaviors to mitigate the risk of LBW.

*“Based on my observation (factors affecting LBW) is passive smoking, in addition to mothers with eating difficulties” (HC8)*

*The [social] environment plays a crucial role. There are fathers smoking within the family, led to poor air quality and disrupts the economy. The family should be able to buy eggs, but in fact for cigarettes. (HC7)*

#### 3.2.5. Unintended Pregnancy

Care providers and mothers have expressed concern that unintended pregnancies are associated with adverse neonatal outcomes. They have noted that such pregnancies can lead to complications like preterm delivery and intrauterine growth retardation, both of which contribute to an increased incidence of LBW. Observations indicate that unintended pregnancies are linked to significantly higher odds of having a LBW baby. Additionally, mothers have reported that unintended pregnancies can negatively impact long-term child development, including growth indicators that fall below recommended levels and increased risk of mortality for children under 5 years of age. The anxiety, depression, and high stress levels experienced by women facing unintended pregnancies are believed to contribute to these adverse outcomes, highlighting the need for targeted support and interventions from healthcare providers to address these challenges effectively.

*She didn't have a husband, got pregnant and deliver a LBW baby. Maybe because from the beginning she didn't know that she was pregnant. We did not expect if the mother was pregnant [from the physical appearance], we even did not know her gestational age at the time of LBW delivery. (HC2)*

#### 3.2.6. Scarcity of Healthy Food Sources

During the interviews, participants expressed concerns about local food as a means for expectant mothers to meet their nutritional needs. While they acknowledged the benefits of accessing fresh, seasonal produce and supporting the local economy, some mothers raised issues regarding the availability and consistency of local food sources. Concerns about the lack of variety and the potential for limited nutritional options were highlighted, particularly in areas where access to diverse foods is restricted. Additionally, participants noted that while local food can help preserve traditional food cultures, there may be gaps in knowledge about proper preparation and nutritional value, which could impact the overall effectiveness of local food in fulfilling their dietary needs during pregnancy.

*At the time meal support is discontinued, they will not confused to discover that source of nutrition is surrounding us (HC1)*

*Local food is more affordable, variative and widely available at posyandu (HC11)*

*Another than [nutritional] values, if we prepare it ourselves, we can manage it better and save more [money] because we cook it at home (HC11)*

The findings were validated by mothers who consume local foods:

“*I have consumed star gooseberry (Sauropus androgynus), steamed bean and corn, bay leaf, malabar spinach” (M5)*

### 3.3. Theme 3: Enhancing the Use of Digital Technology

#### 3.3.1. Sustainability

Care providers have emphasized that program continuity is crucial in health interventions to ensure both effectiveness and sustainability. They highlight that the continuum of care concept, which integrates systems of care and empowers and monitors patients through personalized health services over time, has been shown to improve outcomes in areas such as disease prevention, maternal and child health, and psychological well-being. According to healthcare providers, maintaining continuity in patient care and auxiliary support is fundamental to delivering essential healthcare services. They assert that a solid continuity plan within health organizations is critical not only for service delivery but also for protecting the privacy and safety of patients. By prioritizing continuity planning, healthcare organizations can position themselves to effectively avert threats and recover essential patient care services.

*No existing follow up on how far the level of complaints from mothers about stunting has been done so far, what is it about child nutrition and so on (HC3)*

*Further optimization of assistance for pregnant women who are at risk is necessary (HC3)*

*Later, we monitor (the child growth) from the KMS [Health Monitoring Card], which is on the nutritional status sheet that require mothers to indicate (HC9)*

Mothers expressed that they were actively engaged during prenatal care:

*“..we regularly check up to the PHC and also the midwives visit us (the pregnant mothers)... in addition, infant and toddlers are entitled for supplementary nutritional packages” (M6)*

#### 3.3.2. Feature Developments

Mobile health (m-health) interventions in low- and middle-income countries have shown promise in improving antenatal and postnatal care uptake, skilled birth assistance, and vaccination rates. Participants noted that existing web services need improvement to enhance their effectiveness. While some m-health solutions have been beneficial in increasing women's knowledge and practices regarding infant care, there are gaps in the current services that limit their impact. Enhancements to these platforms could better support pregnant women and new mothers by providing more accessible, tailored information, resources, and guidance, ultimately helping to prevent LBW and improve maternal and child health outcomes.

*We need this feature to allow pregnant mother's report when she gives birth, the baby's weight, and height (HC1)*

*Among the application users, we need to identify those who have given birth, those who are LBW, that's all because the purpose of this system was originally to reduce the incidence of LBW (HC1)*

*(I) expect to monitor my child's growth and development (by using the app) (M11)*

Positive impressions were expressed by the mother:

*“The application is helpful to monitor child's growth” (M7)*

#### 3.3.3. Knowledge Enhancements

Educational health interventions have the potential to transform individual mindsets by customizing activities, methods, and messages to align with the community's preferences. The study revealed that such tailored interventions can effectively promote health-enhancing lifestyles and improve health-related behaviors. Participants emphasized the importance of implementing educational sessions specifically designed for expectant mothers, as these sessions could facilitate a shift toward a growth-oriented mindset. By providing relevant information and support, these educational initiatives can empower mothers, ultimately leading to better health outcomes for both themselves and their infants.

*On average, [educational contents] have been delivered. We educate about breastfeeding, and also with cadres. However, it need to be refreshed, maybe it's about breastfeeding (HC9)*

Mothers are satisfied with the amount of educational sessions:

*I would say that the educational sessions are enough. The midwives taught about breastfeeding during pregnancy classes (M7)*

## 4. Discussion

This study is among the first to examine the burden faced by healthcare providers in managing LBW, particularly within the context of Indonesia, with a specific focus on the Yogyakarta region. Through examining the perspectives of healthcare providers directly involved in LBW programs, the current research offers critical insights into the challenges and complexities of service delivery in Kulon Progo Regency. The findings of this study are organized into three major themes and subsequently discussed in chronological order, which are presented in the following sections.

### 4.1. Programmatic Initiatives of the Healthcare Providers

The first theme captures the orchestrated efforts to address the needs of mothers with LBW infants. This theme encompasses a range of programs implemented at both the community and individual levels, including routine home visits, provision of dietary support, and engagement through integrated health posts (Posyandu). Additionally, private consultations and targeted interventions were employed to deliver personalized care and address specific risk factors.

Those initiatives undertaken by healthcare providers are vital in addressing LBW and enhancing maternal and child health outcomes. The strategies include community outreach programs, educational workshops, and integrated care models tailored to meet the specific needs of mothers and infants [14]. By fostering collaboration among multidisciplinary teams, including midwives, nurses, and community health workers, a comprehensive approach to care is established, essential for effectively managing LBW. However, the success of these programs relies heavily on adequate support, funding, and active engagement from the community.

Targeted health interventions that address the unique needs of expectant mothers are crucial for enhancing maternal care and improving outcomes related to LBW. By encouraging healthy lifestyle choices—such as regular exercise, proper nutrition, and smoking cessation—healthcare providers can offer personalized advice and support to those at risk for pregnancy complications. Furthermore, implementing tailored care plans, comprehensive education, and ongoing support enables more effective delivery of care during pregnancy, ultimately fostering better health and quality of life for mothers and their infants.

Multiple studies have demonstrated that collaborative approaches involving healthcare providers and community members contribute significantly to improving quality of care by generating relevant data for priority setting and accurately identifying local health challenges [15, 16]. Enhancing communication, increasing awareness, and improving service delivery through community visits are anticipated to positively impact population health outcomes. Prior to the implementation of programs, it is essential to assess health and community data to gain a comprehensive understanding of the local context and priorities. Within the health system, various stakeholders—such as volunteers, healthcare providers, and local healthcare facilities—play pivotal roles in fostering connections between the community and health services [17].

Various elements must be taken into account while developing community engagement programs. Enabling factors represented in political will, public awareness, laws, and resources with a consistent and firm understanding of how the programs work are necessary. In addition, it is imperative to use local-context data in planning, monitoring, decision-making, and accountability of the community management. Since the targeted population is diverse, intercultural sensitivity should recognize, respect, and consider local beliefs and practices and understand social networks and norms within the context of the program [18]. Finally, the health system's capacity to interact with communities and broader systems in driving constructive dialog during the program development and implementation is essentially required.

### 4.2. Poor Knowledge, Attitude, and Practice With Limited Resources and Access

The second theme highlights the underlying socioenvironmental and individual factors that hinder effective management of LBW. This theme comprises several interrelated subthemes, including poor nutritional status, limited knowledge about maternal and child health, and the presence of pregnancy complications. Additional contributing factors such as passive smoking and unintended pregnancies further pose a vulnerability of both mothers and infants.

A significant barrier to effective healthcare delivery is the shortage of healthcare providers, particularly in low- and middle-income countries [19]. Insufficient staffing levels and inadequate training severely limit the ability of healthcare providers to provide essential services, especially in remote or underserved areas. This lack not only affects the availability of care but also contributes to disparities in health outcomes, including increased rates of LBW. Geographical barriers, such as distance to healthcare facilities and challenging terrain, further exacerbate the issue, making it difficult for community members to access necessary services. Efforts to recruit, train, and retain healthcare personnel are essential for enhancing maternal care and addressing LBW challenges. A well-trained workforce can provide comprehensive antenatal and postnatal services, ensuring timely interventions that mitigate LBW risks. Additionally, improving healthcare infrastructure increases access to essential services, enabling expectant mothers to engage in preventive care and health education critical for optimal maternal and child health outcomes [6].

Collaboration between communities and healthcare providers in delivering care is often hindered by significant challenges. Geographical barriers, such as distance, difficult terrain, and inadequate transportation options, limit access to healthcare facilities for community members [17, 20]. As a result, healthcare providers face significant challenges in executing effective outreach programs. Furthermore, both community members and healthcare providers may be hindered in their collaborative efforts due to inadequate communication skills, constrained financial and technical resources, and policies that lack support for integrated care delivery systems. These elements lead to a fragmented approach to healthcare, which ultimately undermines the effectiveness of care provision and adversely affects overall health outcomes within the community.

Numerous enabling and impeding elements have been identified concerning community capacity development, both in terms of leadership skills, governance, and management. Health literacy and behaviors particularly determine the community health status. Participatory programs involving stakeholders and community members require intense engagement to ensure the effectiveness of the health intervention [16, 21]. Poor resource management and decision-making transparency undermined the confidence required for successful provider–community cooperation.

### 4.3. Enhancing the Use of Digital Technology

The third theme reflects emerging efforts to leverage technological solutions in addressing the challenges associated with LBW management. This theme includes sustainability for the initiatives, the development of new features based on user needs, and enhancing users' knowledge through digital platforms. Digital tools have the potential to support healthcare providers, improve maternal and neonatal outcomes, and strengthen the overall health system in resource-limited settings.

The integration of digital technology into healthcare systems has the potential to transform the management of LBW by improving access to care and facilitating better communication between providers and patients. m-health interventions, telemedicine, and electronic health records enhance the delivery of antenatal and postnatal care, enabling healthcare providers to monitor patient progress and provide personalized support more effectively [22–24]. These technological advancements allow for the dissemination of crucial health information, promote patient engagement, and empower expectant mothers to actively manage their health. By leveraging digital tools, healthcare systems can overcome geographical barriers and enhance the effectiveness of outreach programs, ultimately leading to improved maternal and child health outcomes in the context of LBW prevention [24, 25].

Nevertheless, the sustainability of the digital initiatives requires careful planning to ensure long-term effectiveness and integration within the broader health system. Key considerations include the alignment of digital tools with existing healthcare facility information systems and electronic medical records, as well as the establishment of procedures to support continuity of care from community-based outreach to facility-based services [26, 27]. Additionally, extending the scope of care beyond pregnancy and early childhood is essential to address the ongoing needs of mothers and children, thereby enhancing the overall impact and sustainability of LBW interventions.

The next critical concern to address is the maximization of digital technology utilization within LBW management programs. Effective implementation depends on a user-centered design approach that ensures the tools are accessible, relevant, and responsive to the needs of both healthcare providers and mothers. Capacity building, including training for both trainers and end-users, to foster digital literacy and confident engagement with the systems is also necessary [28, 29]. Continuing utilization also requires the provision of both technical and nontechnical support, alongside attention to infrastructure-related factors such as network coverage, particularly in rural areas.

A notable gap in the availability of digital resources specifically designed to address LBW is observed. While several digital health tools exist for general maternal care, few focus on the prevention and management of LBW. The current study highlights the lack of customized applications that can provide personalized care and timely interventions for mothers with risk for LBW. Key concerns raised include the need for digital platforms that offer real-time monitoring, tailored health education, and comprehensive management of risk factors [30].

In relation to knowledge enhancement, further study is necessary to assess users' preferences in learning to ensure that digital interventions are both effective and engaging. The findings of this study indicate the need for diversification of content, tailored delivery methods, and context-appropriate engagement strategies to meet the varied learning needs of users. Moreover, incorporating structured knowledge evaluation mechanisms will be essential to measure the effectiveness of educational components and to continuously refine the digital learning experience [31–33]. These considerations are vital for optimizing the role of digital platforms in enhancing provider and community knowledge related to LBW management.

### 4.4. Limitation of the Study

This study acknowledges several limitations that may impact its findings. One notable constraint is the location of data collection was in working area of one public health center in Kulon Progo District, which may not adequately represent the diverse background of the healthcare providers and mothers. It is suggested that future research should focus on a larger area that would enhance the study's ability to capture a broader spectrum and provide deeper insights into the complexities faced by healthcare providers regarding LBW.

## 5. Conclusion

Addressing LBW effectively requires leveraging advancements in technology and telecommunication to enhance programmatic interventions led by healthcare providers. By integrating digital tools, we can improve communication channels, expand outreach efforts, and provide timely support to both mothers and healthcare providers. This technological integration not only strengthens the availability and accessibility of health human resources but also optimizes care delivery in resource-limited settings. Emphasizing these innovations within program interventions offers a promising pathway to better manage LBW and improve maternal and child health outcomes.

## Figures and Tables

**Table 1:** Midwife participant characteristics.

**Participant ID**	**Age (years)**	**Educational background**	**Years of experience**
HC2	54	Midwife (3-year diploma)	33
HC3	47	Midwife (3-year diploma)	26
HC4	48	Midwife (3-year diploma)	26
HC5	43	Midwife (3-year diploma)	25
HC6	46	Midwife (4-year diploma)	16
HC7	48	Midwife (3-year diploma)	18
HC8	37	Midwife (4-year diploma)	15
HC9	39	Midwife (3-year diploma)	15
HC10	44	Midwife (3-year diploma)	18

**Table 2:** Healthcare providers in triangulation.

**ID**	**Age**	**Educational background**	**Years of experience**
HC1	51	MD, management	8 years
HC11	45	Nutritionist	30 years

**Table 3:** Characteristics of mothers as source of triangulation.

**ID**	**Age**	**Educational background**
M1	28	Senior high school
M2	31	Senior high school
M3	27	Senior high school
M4	28	Senior high school
M5	23	Senior high school
M6	16	Junior high school
M7	27	Senior high school
M8	30	Senior high school
M9	20	Senior high school
M10	31	Senior high school
M11	38	Senior high school
M12	40	Bachelor

**Table 4:** Codes, subthemes, and themes.

**Code**	**Subtheme**	**Theme**
Postnatal care	Home visits	Programmatic initiatives of the healthcare providers
Meal supplements	Dietary support	
Out-facility programs, weight monitoring	Integrated health post	
Ask the midwives	Private consultations	
	Targeted interventions	
Eating difficulties, dietary pattern	Poor mothers' nutritional status	Poor knowledge, attitude, and practice with limited resources and access
Parenting style, educational background	Lack of knowledge	
Prematurity, multiple pregnancy, preeclampsia	Pregnancy complications	
Fathers/husband smoking	Passive smoking	
Premarital pregnancy	Unintended pregnancy	
Longitudinal data	Sustainability	Enhancing the use of teleconsultations
Self-reporting, catered health articles	Feature developments	
Deliver educational contents	Knowledge enhancements	
Focus on children at risk		

## Data Availability

The data that support the findings of this study are available from the corresponding author upon reasonable request.
